# Acute Myeloid Leukemia in a Patient With Xeroderma Pigmentosum: A Case Report

**DOI:** 10.1002/ccr3.73260

**Published:** 2026-07-28

**Authors:** Mina Soryal, Mohamed Mahmoud Marey, Malak A. Hassan, Rawan El‐Sayed, Enas Kamal Abdalaziz Seyed, Ashraf Elghandour

**Affiliations:** ^1^ Department of Internal Medicine, Faculty of Medicine Alexandria University Alexandria Egypt; ^2^ Faculty of Medicine Alexandria University Alexandria Egypt; ^3^ Faculty of Medicine Al‐Neelain University Khartoum Sudan

**Keywords:** acute myeloid leukemia, case report, hematological malignancy, xeroderma pigmentosum

## Abstract

Acute myeloid leukemia may occur in patients with xeroderma pigmentosum at a younger age and can be associated with complex cytogenetics and poor treatment response, highlighting the importance of regular hematologic monitoring.

## 1 Introduction

Xeroderma pigmentosum (XP) is a rare autosomal recessive disorder that exhibits unique geographic variation. It affects 1 per 1 million people in the United States, 2.3 per 1 million people in Western Europe, and 45 per 1 million people in Japan [1]. The condition has also been reported throughout the Middle East and North Africa, where larger families and high rates of consanguinity exist [1]. Moriz Kaposi's initial description of XP was based on four patients with thin, dry skin, wrinkles, checkered pigmentation, tiny vascular dilatations, skin contraction, and cutaneous malignancies [2].

The pathogenesis of XP is defective nucleotide excision repair, preventing cells from eliminating UV‐induced photoproducts and repairing DNA damage [3]. Mutations in the bypass polymerase, POLH/XPV, or in the DNA repair/transcription genes, XPA, ERCC3/XPB, XPC, ERCC2/XPD, DDB2/XPE, ERCC4/XPF/FANCQ, or ERCC5/XPG, have been found to contribute to XP development [4]. Thus, genetic testing for mutations in the nucleotide excision repair (NER) gene and cellular assays for UV sensitivity confirm the diagnosis. Freckles and pigmentary changes before age two are common symptoms of XP [4]. Clinical manifestations include eye damage, severe photosensitivity, and early‐onset skin malignancies [4]. The management of XP includes lifetime UV protection, vitamin D supplementation, and drugs such as isotretinoin [4]. Despite this, XP patients remain at high risk for various malignancies.

Skin malignancies in XP are common, aggressive, and manifest in early childhood [4]. Patients frequently develop basal cell carcinoma, squamous cell carcinoma, and melanoma [4]. The median age at initial diagnosis is 9 years for non‐melanoma skin cancers and 22 years for melanoma, in contrast to 67 years and 55 years, respectively, in a healthy population [4]. More than 80% of non‐melanoma skin cancers arise in sun‐exposed areas such as the face, head, and neck [5, 6, 7, 8]. Central nervous system (CNS) malignancies have been reported in XP patients, too, with an estimated 10‐ to 20‐fold increase in incidence compared to healthy individuals [5, 6]. In addition, there have been prior reports of isolated cases of hematological malignancies like acute myeloid leukemia (AML), acute lymphoid leukemia (ALL), and/or myelodysplastic syndrome (MDS) in XP patients [5, 9].

AML is a rapidly progressing myeloid neoplasm characterized by clonal expansion of immature myeloid cells, known as blasts, causing ineffective erythropoiesis, ineffective megakaryopoiesis, and bone marrow failure. Although the exact cause is frequently unknown, genetic mutations and previous exposure to benzene, radiation, and chemotherapy are implicated in its etiology. Patients typically present with pallor, infections, bleeding, and fatigue. Complete Blood Count (CBC), bone marrow aspiration (BMA), blood smear (featuring blasts with Auer rods), and immunophenotyping are all utilized in diagnosis. Cytarabine‐anthracycline chemotherapy is part of the treatment, and in certain circumstances, stem cell transplantation is also an option [6].

We report a case of AML in an Egyptian male with XP since childhood, adding to the limited but growing literature documenting this rare association. The predominance of dermatological manifestations in XP patients may overshadow or delay the recognition of serious hematologic conditions like AML. Thus, our study highlights the importance of vigilant hematologic monitoring in XP patients.

## 2 Case Presentation

### 2.1 Case History/Examination

A 38‐year‐old Egyptian male patient presented to Alexandria Main University Hospital in May 2024 with epistaxis, bone aches, fever, night sweats, and weight loss for 3 months. The patient's past medical history included XP since childhood. He suffered from burning on minimal sun exposure, causing significant limitations to his daily activities. His physical and mental development was normal. His neurological examination, including hearing assessment, was normal. Bilateral corneal ulcers and multiple benign skin tumors complicated his XP. The skin tumors had been surgically removed, and skin grafting was done. In addition, clinical examination revealed hyperpigmented cutaneous lesions, crusts, and freckles on sun‐exposed areas. No organomegaly or palpable lymphadenopathy was detected. The patient had no other chronic diseases. The family history was negative for XP and hematological disorders.

### 2.2 Differential Diagnosis, Investigations, and Treatment

As a part of the initial assessment, a CBC revealed normocytic normochromic anemia, thrombocytopenia, and leukocytosis. Notably, there was an Absolute Neutrophil Count (ANC) of 0.8 G/L and 60% blast cells. More details regarding the patient's CBC on admission are provided in Table 1. Bone marrow aspiration (BMA) and flow cytometry were done. BMA revealed 53% blast cells, with features suggestive of Myelodysplastic Syndrome. The patient tested positive for the following CD markers: CD13, CD33, CD34, CD7, CD45 (dim), HLA‐DR, and CD19 (partial). The patient tested negative for the following CD markers: CD10, CD2, CytCD3, and CytCD79a. A diagnosis of AML with aberrant CD19 on top of a pre‐existing myelodysplastic neoplasm was established.

**TABLE 1:** Complete blood count at the time of admission.

Parameter	Result	Reference range
Hemoglobin	4.7 g/dL	(13–17)
Hematocrit	15.2%	(40–50)
Mean corpuscular volume	90.3 fL	(83–101)
Mean corpuscular hemoglobin	28.0 pg	(27–32)
Mean corpuscular hemoglobin concentration	31.0 g/dL	(31.5–34.5)
Red blood cells	1.69 × 10^6^/μL	(4.5–5.5)
Platelets count	13 × 10^3^/μL	(150–410)
White blood cells	17.82 × 10^3^/μL	(4–10)
Basophil	0%	(0–2)
Eosinophil	1%	(1–6)
Neutrophil	5%	(40–75)
Lymphocyte	29%	(20–45)
Monocyte	5%	(2–10)
Blast cells	60%	

**FIGURE 1:**
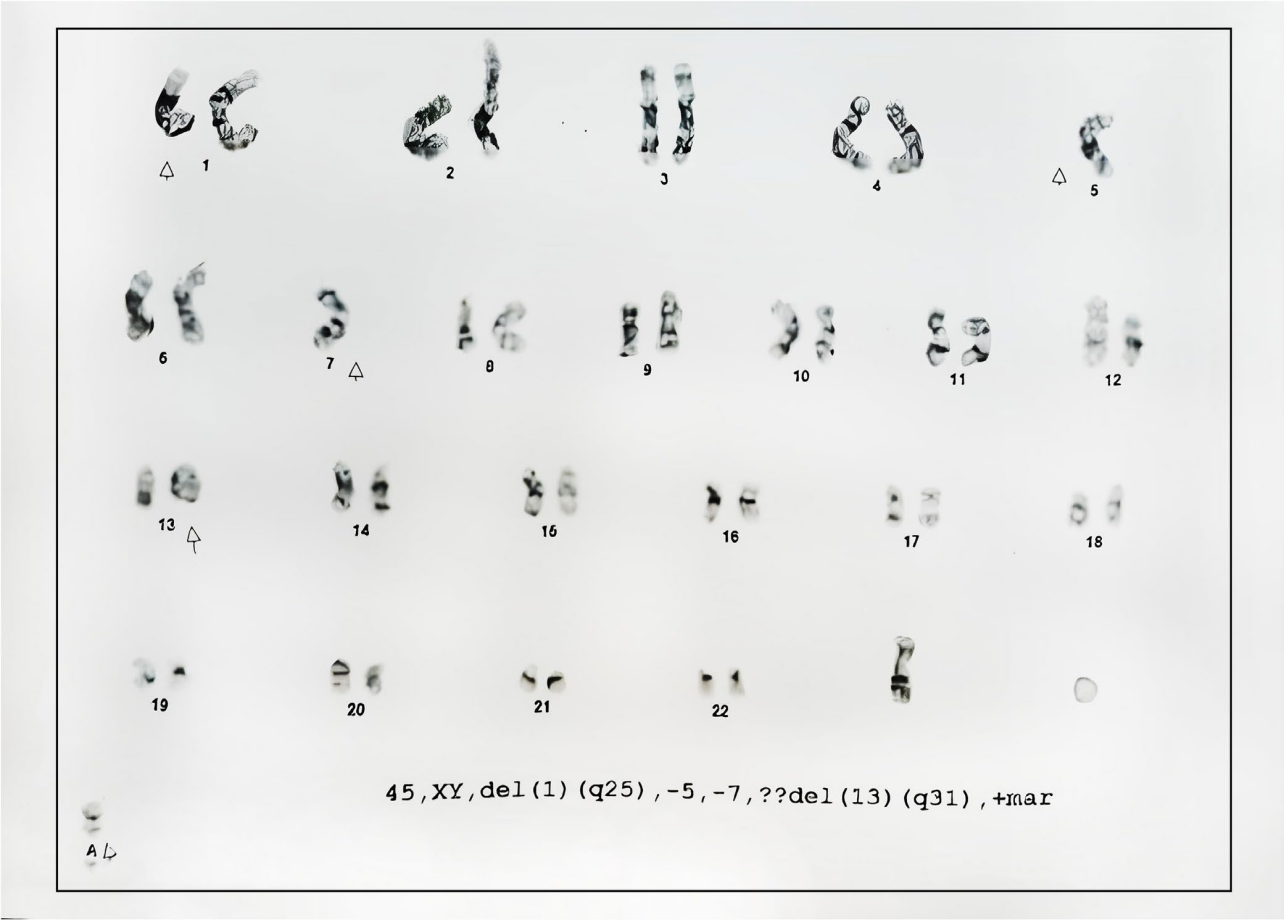
Cytogenetic analysis (Karyotype). Karyotype showing a complex chromosomal profile including deletion in the long arm of chromosome 1 [del(1)(q25)], loss of chromosomes 5 and 7 (−5, −7), deletion in the long arm of chromosome 13 [del(13)(q31)], and an unidentified marker chromosome (+mar).

The patient tested negative for FLT3 mutation. A chromosomal breakage test was also done to exclude hereditary syndromes such as Fanconi anemia. The chromosome breakage test was negative for both induced and spontaneous breakage. Karyotyping, as shown in Figure 1, showed a deletion in the long arm of chromosome 1, loss of one copy of chromosomes 5 and 7, a deletion in the long arm of chromosome 13, and an additional chromosome that could not be identified.

### 2.3 Outcomes and Follow‐Up

The patient was started on the 7 + 3 regimen with cytarabine 100 mg/m^2^ continuous infusion for 7 days and daunorubicin 60 mg on days 1, 3, and 5. During the induction treatment, the patient developed severe febrile neutropenia, pneumonia, and septicemia. Unfortunately, the patient did not respond to conventional chemotherapy. Follow‐up BMA 5 weeks later showed 68% blast cells, an increase from the pre‐treatment BMA.

The patient was not fit to receive another intensive chemotherapy. Therefore, the decision was made to start treatment with subcutaneous cytarabine 100 mg for 7 days monthly with oral mercaptopurine daily. However, the patient did not respond to treatment, and his laboratory investigations continued to feature progressive leukocytosis, anemia, and thrombocytopenia. Two months later, the patient was readmitted with severe sepsis and intra‐alveolar hemorrhage. Unfortunately, the patient died 2 weeks after the second admission due to septic shock.

## 3 Discussion

This case report features a rare case of AML in a 38‐year‐old patient with XP. The patient failed to respond to conventional chemotherapy. He later passed away due to the complications of AML.

Cutaneous malignancies and neurodegeneration are well‐recognized complications of XP [3]. In fact, cutaneous malignancies are the most common cause of death in XP patients, followed by neurodegeneration [3]. Other non‐skin malignancies that are more prevalent in XP patients include CNS, ocular, and lung tumors [3]. Hematological neoplasms, such as AML, have not been thoroughly explored or frequently reported in patients with XP. Thus, this case report documents an interesting case of AML in a patient with XP. Only two previously published case reports of AML in patients with XP exist [9, 10], as well as a case report of mixed‐phenotype acute leukemia (MPAL) in a patient with XP [6].

Bencharef et al. reported a case of AML with complex karyotype and monosomy 7 in a 26‐year old Moroccan patient who presented with anemic and hemorrhagic symptoms [10]. The patient was treated with a low‐dose cytarabine protocol and granulocyte colony‐stimulating factor [10]. However, the patient did not tolerate the chemotherapy and passed away due to acute pulmonary edema and cardiac tamponade [10].

Janjetovic et al. documented a case of AML with complex karyotype in a 33‐year old male XP patient [9]. He was treated with chemotherapy, to which he responded and achieved complete remission [9]. He subsequently underwent allogeneic stem cell transplantation [9]. Unfortunately, he passed away a month and a half later due to AML relapse and sepsis [9].

In a letter to the editor, Pintens et al. refer to two Moroccan sisters with XP [11]. One developed AML and subsequently passed away at the age of 28, and the other developed ALL and subsequently passed away at the age of 25 [11]. Though limited data on the cases are provided, their observation suggests that the pathogenesis in the development of both hematological malignancies in XP patients may be similar.

Oetjen et al. described a case of mixed‐phenotype acute leukemia (MPAL) in a female Moroccan patient with a homozygous North African XPC founder mutation [6]. The patient had an extensive history of cutaneous malignancies [6]. She presented at the age of 19 with night sweats and lymphadenopathy [6]. Initial lab work revealed pancytopenia with 18.2% blast cells, and further investigations confirmed MPAL [6]. The patient responded to treatment and achieved complete remission [6].

To the best of our knowledge, our case study presents the third documented case in the literature of AML in a patient with XP, highlighting the rarity of this association and the significance of this case report. Interestingly, similarly to our patient, most of the previously discussed cases were from the North African region. This raises questions surrounding the geographical distribution of this association. Further prospective studies are needed to assess the mutations underlying XP in North African patients and how they correlate with the disease prognosis and complications, particularly hematological malignancies.

The median age of onset of AML is 68 years [11]. All the previously reported cases of AML in patients with XP, as well as our case, feature drastically younger patients [9, 10, 11]. Our patient exhibited a poor response to chemotherapy. This was also seen in the cases described by Bencharef et al. and Pintens et al. [10, 11]. Although the case described by Janjevotci et al. initially featured complete remission, it later relapsed [9]. Complete remission was achieved in the case described by Oetjen et al., though it is worth noting that the patient's leukemia was of a mixed‐phenotype [6]. Further studies are needed to further explore chemotherapy response and chemotherapy intolerance in patients with XP.

Though typically the most common causes of death in patients with XP are cutaneous malignancies and neurodegeneration, our patient, as well as the majority of the cases previously discussed, had passed away as a result of AML and its complications. This suggests that this association may carry a poor prognosis.

The pathogenesis underlying this potential association remains unclear. Previous studies have explored the polymorphisms of DNA repair genes and how they correlate to leukemia risk [12, 13, 14, 15]. Interestingly, a case–control study conducted in Egypt found that patients who were homozygous for XPD, either alone or in combination with variant genotypes of XPC and XPG, had a fourfold chance of developing AML [16]. This suggests that XPD polymorphism may serve as a molecular marker linked to an increased risk of developing AML [16, 17]. Our interpretation here is limited by the lack of specific XP mutation data for this patient.

It is critical for physicians who encounter this association to thoroughly document it so that we may identify patterns in the demographics, clinical presentation, and response to therapy. In doing so, we can improve the management of patients who present with this association in the future.

## 4 Conclusion

Though cutaneous malignancies are extremely prevalent in patients with XP, hematological malignancies such as AML have not been frequently reported in patients with XP. To the best of our knowledge, this case report is the third in the literature to discuss AML in patients with XP. More case reports and observational studies are needed to further explore the occurrence of hematological malignancies in patients with xeroderma pigmentosum. Furthermore, the geographical distribution of such an association may offer further insight.

## Author Contributions

**Mina Soryal:** project administration, writing – original draft, writing – review and editing. **Mohamed Mahmoud Marey:** writing – original draft, writing – review and editing. **Malak A. Hassan:** writing – original draft, writing – review and editing. **Rawan El‐Sayed:** writing – original draft. **Enas Kamal Abdalaziz Seyed:** writing – original draft, writing – review and editing. **Ashraf Elghandour:** supervision, writing – review and editing.

## Funding

The authors have nothing to report.

## Ethics Statement

Obtaining ethical approval was waived in accordance with the institution's guidelines.

## Consent

Written informed consent for publication of this case report and any accompanying images was obtained from the patient. A copy of the signed consent form is available for review.

## Conflicts of Interest

The authors declare no conflicts of interest.

## Data Availability

Clinical and laboratory data are available.
